# A frugal arduino-based spirometer for low-resource settings: design, development and validation of a preliminary prototype

**DOI:** 10.3389/fbioe.2025.1664127

**Published:** 2026-01-06

**Authors:** James Wallace, Pedro Checa Rifá, Thanusan Kannathasan, Charles F. Hayfron-Benjamin, Philip Anyanwu, Davide Piaggio

**Affiliations:** 1 School of Engineering, University of Warwick, Coventry, United Kingdom; 2 Departments of Physiology and Anaesthesia/Intensive Care, University of Ghana Medical School, Accra, Ghana; 3 Warwick Medical School, University of Warwick, Coventry, United Kingdom

**Keywords:** asthma, COPD, frugal innovation, low- and middle-income countries, low-resource settings, medical device, spirometry, respiratory diseases

## Abstract

**Background:**

Medical devices are essential for maintaining resilient health systems worldwide. However, their distribution does not reflect this, with as many as 76% of devices being used by 13% of the world’s population. Most devices, being designed for High-Income countries (HICs) and failing to consider the local needs of other settings, often fail when deployed in other contexts. Spirometers particularly are underutilised in these regions despite high respiratory disease burden. This study aims to develop and validate a frugal affordable spirometer for low-resource settings (LRSs).

**Methods:**

A Venturi-style spirometer was designed using 3D printed components, low-cost electronics such as the Arduino nano (ESP32) and differential pressure sensor (MXP5010DP), and software such as MATLAB and Arduino IDE. The device was validated through a 2-L pump test and a prospective evaluation study involving 30 participants, comparing its performance to a CE marked spirometer. Key metrics included were the mean absolute error (MAE), mean absolute percentage error (MAPE), Pearsons’s correlation, and Intraclass correlation coefficients (ICC).

**Results:**

The device was successfully 3D printed from Polylactic acid (PLA) using an Ultimaker 2+ printer and assembled with electronic components. The device achieved a MAP error of 1.53% in the pump test. In the prospective evaluation study, the device showed strong agreement for forced expiratory volume in 1 s (FEV1) with the benchmark device (ICC = 0.97, r = 0.97, p < 0.001). Peak expiratory flow (PEF) measurements were less accurate (MAPE = 18.01%) but still demonstrated strong correlation (ICC = 0.80, r = 0.89). This accuracy was in line with the International Standards Organisation 26782:2009 standards for accurately measuring volume.

**Conclusion:**

This study designed, prototyped and validated a spirometer that can be used as a lung screening tool, that achieves high accuracies comparable to those of other portable spirometers. This study also validated that frugal engineering could reduce the cost of devices without affecting the clinical accuracy. Whilst promising, further validation with clinical populations and better alignment with regulatory standards are needed before use in LRSs.

## Introduction

Medical devices and health technologies are crucial in building resilient and effective health systems. They can enhance diagnosis and treatment, improve patient safety and care quality, and boost efficiency and productivity. Modern medical devices increasingly generate data and integrate with consumer technologies, expanding their capabilities (WHO, 2017). These technologies are used across a wide range of functions—from diagnosing and monitoring conditions to supporting individuals with disabilities and treating both acute and chronic illnesses. However, the distribution and use of medical devices remain highly unequal.

Currently, the global market is estimated to have two million different types of medical devices grouped into 7,000 generic categories (WHO, 2017). HICs, representing just 13% of the global population, account for over 75% of this medical device usage (Anner, 2013), whilst low- and middle- income countries (LMICs) mainly rely on medical device donations. It is estimated that 80% of medical devices are donated by international and nongovernmental organisations in LMICs, but of these donated devices only 10%-30% are operational (WHO, 2010), largely because they often fail to meet the contextualised needs of recipient countries. Among the different challenges there often are inappropriate technical specifications, lack of supporting infrastructure, or insufficient staff training (Ongaro et al., 2022; WHO, 2024a). This heavy reliance on external donations can also compromise long-term health system sustainability by creating supply chain vulnerabilities, hindering the development of local capacity for innovation and maintenance, and reducing autonomy in healthcare planning and delivery (Ibrahim et al., 2025). Additionally, the anticipated global population growth, particularly in LMICs (Leong et al., 2019), underscores the need for proportional universalism in the development and distribution of medical devices, ensuring increased availability and contextually tailored designs in regions with the highest need.

These disparities in medical devices are particularly evident in the management of respiratory diseases, which disproportionately affect LMICs (Vos et al., 2020). For example, lower respiratory tract infections were the leading cause of death in LICs in 2021 (WHO, 2024b), and over 90% of chronic obstructive pulmonary disease (COPD) deaths among individuals under 70 occurred in LMICs (WHO, 2013). Similarly, 96% of global asthma deaths in 2019 were concentrated in these regions (GBDCRD Collaborators, 2023). Despite the high burden, access to appropriate diagnostic tools remains limited, contributing to underdiagnosis and mismanagement. Increasing access to affordable and context-specific medical devices in LMIC settings could help narrow regional disparities (Yadav et al., 2021).

The most commonly used devices for diagnosing respiratory diseases include peak flow meters, spirometers and pulse oximeters (Thomas et al., 2000). Among these, spirometers are the most versatile, offering a more comprehensive assessment of lung function by generating full volume time curves, rather than isolated values such as peak flow. They primarily measure key indicators such as forced expiratory volume in 1 s (FEV1), forced vital capacity (FVC) and peak expiratory flow (PEF), providing the most reliable data for differentiating between conditions such as COPD and asthma (Moore, 2012). Despite their diagnostic value, spirometers remain underutilised in low-resource settings (LRS) because of relatively high costs and the need for trained personnel to operate and interpret the results (Vanjare et al., 2016).

Frugal innovation offers a promising pathway to address these challenges. By focusing on affordability, simplicity and contextual relevance, frugal design principles aim to create medical devices that are accessible and sustainable in resource constrained environments (Tran and Ravaud, 2016; Montesinos et al., 2024). This approach is proven to improve the cost effectiveness and reliability of solutions for the constraints of LRS (Piaggio et al., 2021a), shown in these specific examples (Piaggio et al., 2021b; Williams et al., 2022; Piaggio et al., 2024a; Piaggio et al., 2024b).

### Literature

Despite the growing availability of spirometry devices, several studies highlight the persistent limitations that hinder their uptake in LRS. Carpenter et al. (2018) reviewed 16 commercially available portable spirometers (priced $99–$1,390) and found that all the devices showed limited information on data security, accuracy, and patient outcomes. The paper further reported that whilst all the devices could measure basic lung function parameters, only 43.8% provide immediate feedback on the manoeuvre quality to the user and only 31.3% included instructional videos in their app to guide this. This lack of guidance further increases the chances of wrong use of the device in LRSs, where there may be a lack of dedicated training to healthcare workers and end users. Furthermore, only 50% of the studies included in the review by Carpenter et al. reported their devices accuracy, while none reported their patient outcome data.

Similarly, Wu et al. (2022) ran a technical performance analysis of 10 portable spirometers using International Standards Organisation (ISO) standard flow/volume simulations. Of the devices they tested only 30% met all the performance criteria required for spirometry devices, the rest being only partially compliant. This variability in performance, can cause concern in LRS, where funding is limited and the device diagnostic reliability with minimal calibration and maintenance is key.

Furthermore, Zhou et al. (2022) performed a meta-analysis of portable spirometers for COPD diagnosis, reporting pooled sensitivity and specificity of 85% each, with an Area Under Curve (AUC) of 0.91. Despite these high values, there was a notable difference within the results. Such difference was dependant on the setting and the operator expertise. In the same paper Zhou et al. (2022), in fact, reported that portable spirometers used by technicians in tertiary hospitals were significantly more accurate (statistically significant p < 0.05) than desktop spirometers used by trained technicians in primary care settings. This supports the need for devices that are intuitive and easy to use, self-guiding and designed for use outside of specialist professionals.

Ferreira Nunes et al. (2024) recently presented a Venturi based spirometer, using a 3D printed tube and a Honeywell SSCMRRN005PDAA3 differential pressure sensor. The device showed excellent correlation with gold standard spirometry (Intraclass correlation coefficients (ICC) = 0.987 for FVC), validating its accuracy. Whilst it showed high technical performance, several aspects of its development make it unsuitable for LRS. One of the main barriers to these settings is often cost and their device, although highly accurate, relied on expensive components. The cost of the sensor alone was £74.17 and the board used to process the data generally varies from £88–131 depending on the accessories that come with the board (available through direct quote only). This level of cost places the device in a too high-cost tier for its widespread deployment in LMICs. The device further experienced shortcomings such as a lack of an onboard display, limited environmental robustness (open ports, for example, could allow dust intrusion) and limited usability testing, as it was only tested with eight healthy individuals, with no further assessment of its usability in clinical or community settings.

Overall, these findings underscore the urgent need for frugal, user-centred designs that improve the affordability, durability, and usability of medical devices in LRS, paving the way for more accessible healthcare technologies.

### Aims

As presented in the introduction, there remains a clear gap in the availability of spirometers that are not only affordable and accurate, but also designed with the end users and context of use in mind.

Our research question, therefore, was: *Can an affordable and durable Arduino-based spirometer be designed to meet clinical usability and diagnostic requirements in LRS?*

Adopting a frugal innovation approach, we leveraged modern technologies such as 3D-printing and affordable electronics to create a cost-efficient solution. Our design is grounded in a user-centred methodology, prioritising the needs of primary care physicians to ensure accuracy, contextual relevance, ease of use, and broad accessibility. We focus on LRS in LMICs, particularly sub-Saharan Africa, but recognise the potential for use in low-resource healthcare systems of HICs.

## Methods

This section details the methods used to design, prototype, and validate the hardware and software components of the spirometer.

### Hardware

#### Contextualised design

Building on prior research and expertise in designing medical devices for LRSs (Ibrahim et al., 2025; Piaggio et al., 2021a) (and the examples mentioned in the introduction), the spirometer was developed with a user-driven approach, specifically tailored to the needs and constraints of such environments. Two focus groups were conducted to consult 10 biomedical engineering and medicine students from both HICs and LMICs, to inform the co-creation of this device. This was supported by discussions with respiratory clinicians from LMICs who use spirometry to assess patients with respiratory conditions. Key decisions from these consultations included adopting a differential pressure sensing system to eliminate the need for moving parts, such as turbines, thereby increasing the device’s durability and suitability for LRS. While turbine flow and rolling seal systems were considered, they were ultimately dropped due to their fragility and susceptibility to dust and humidity. Another important decision from the consultation was to minimise the number of components and ensure that most parts could be locally sourced or produced *via* 3D-printing. Finally, the design prioritised ease of use and maintenance to enhance usability in diverse settings.

The device is made up of the following key components:

- A 3D-printed Venturi tube to generate the pressure difference;
- A 3D-printed casing that ensures airtight seals for the device and locks together to protect components;
- An electronic board comprising of an Arduino nano (ESP32), differential pressure NXP sensor (model number: MPX5010DP) and indicator electronics such as light emitting diodes (LEDs) and a liquid crystal display (LCD).

#### Tube design

A custom tube, designed using Autodesk Fusion 360, is based on Bernoulli’s principle (Igwe Johnson, 2023), incorporating two distinct internal cross-sectional areas, so that when air flows through it, a differential pressure is created at two points, relative to the different cross-sectional areas. This differential pressure is correlated with the to a flow rate, as per Equation 1 (Igwe Johnson, 2023).

$$Q=A_{1}\sqrt{\frac{2\left(p_{1}-p_{2}\right)}{\rho \left(1-{\left(\frac{A_{1}}{A_{2}}\right)}^{2}\right)}}$$ (1)

Where:

- *Q is the volumetric flow rate (m* ^*3*^ */s),*
- *A*_*1*_ *and A*_*2*_ *are the cross-sectional areas of the cylindrical tube at the level of two different ports (m* ^*2*^ *),*
- *p*_*1*_ *and p*_*2*_ *are the pressures at the same points (Pa), and*
- *rho is the density of air, set at 1.204 kg/m* ^*3*^ *under standard conditions (i.e., 20 °C, 101.325 kPa)* (Caretto, 2008).

Based on Equation 1, the two inner diameters of the tube can be found in order to work efficiently with the selected pressure sensor, namely, MXP5010DP, which has a measurement range of 0–10 kPa. The correct design of this would lead to reduced error and better resolution, reducing the amount of pre- and post-processing needed. For this reason, it was deemed essential to ensure that the maximum pressure difference effectively present between the two ports of the tube was as compatible as possible with the range of the sensor, avoiding situations in which the sensor could either be damaged (over pressure) or could give out readings largely affected by the accuracy. For this purpose, factors such as the maximum user PEF, disposable mouthpiece size, and measurement range of the sensor were considered.

Another key requirement was the minimum internal diameter of the Venturi tube. The diameter should not be too small to avoid creating a large air flow resistance during exhalation, making the test uncomfortable and affecting measurement accuracy. In addition, the chosen transition between the two sections needed to be as smooth as possible in order to minimise turbulence as much as possible. In fact, turbulent flow introduced by non-smooth transitions could increase the noise in the sensor and the perceived resistance, affecting the accuracy of the measurements. For this reason the Venturi Tube specifications from the British Standards Institute (Boyes, 2009) were followed to abide by this as much as possible. In particular three main parameters were used: first, the diameter of the throat/restrictive part should not be less than 0.224D (D being the entrance diameter) of the entrance diameter; the conical converging section should have a taper of 10 ° ± 5 °; and finally, the device should have a divergent outlet of not less than 5 ° and have a minimum length of 1.5 times the throat diameter. All these factors are key to ensuring high accuracy.

Another key characterisation needed was to understand the type of flow within the tube. In order to do this, the Reynolds number was calculated for different airflow velocities to determine the transition flow rate for our diameters (Samantaray and Das, 2019). Reynolds number was calculated using Equation 2, assuming standard conditions:

$$Re=\left(\rho \times v\times d\right)/\mu$$ (2)

Where:

- Re = Reynolds number
-
ρ = density of air (1.204 kg/m^3^)
- v = Velocity of air (m/s)
- d = internal diameter of the tube (0.0278 and 0.0098 m)
-
μ = viscosity of air (1.81 × 10^−5^Kg/(m*s))

For each diameter, a custom code was run on MATLAB to determine the minimum volumetric flow rate to transition air from laminar flow to turbulent flow (when the Re value exceeded 2,000). The code ran volumetric flow rates from 0 to 50 L/min, in steps of 0.1 L/min. When using the larger diameter of 0.0278 m the transition occurred at 39.2 L/min and when using the smaller diameter of 0.0098 m, the transition occurred at 13.9 L/min. Therefore, based on standard human values (peak flow rate of 600 L/min), we could conclude the flow rate would be primarily turbulent. In order to mitigate this, drag equations were taken into account to find the true flow rate from the individual forced exhalation (Colebrook, 1939).

The limits of laminar flow (i.e., the transition zones presented above) were in any case comparable to the noise that would be generated from the sensor, therefore, the laminar drag effects on final volumes could be considered negligible.

#### Material selection

For the physical design three 3D printable plastics were considered:

- Polylactic Acid (PLA)
- Polyethylene Terephthalate Glycol (PETG)
- Acrylonitrile Butadiene Styrene (ABS)

These materials were assessed for their suitability within the spirometry based on multiple criteria. The device firstly should be manufactured out of a low-cost readily available material so that it can be accessible universally. It should not require specialist 3D printers to use nor have too many common issues that could affect the reliability of the print. It should be possible to sterilise the device after repeated use, to ensure hygiene standards are followed. Finally, the device should be durable so that it can withstand significant wear and tear or potential misuse of the device.

Based on these criteria, PLA was chosen to develop the prototype. It offers good rigidity and is dimensionally stable under normal conditions. Furthermore, in terms of availability, it is a very low-cost and accessible material, it does not require specialist printers and has a high-quality surface finish and minimal known printing issues, making it especially suitable for the Venturi tube, which operates best with smooth surfaces.

For implementation in LMICs, PETG would be the most suitable material as it has a high durability, it is recyclable, has better thermal stability than PLA so could withstand low temperature better and maintains mechanical integrity after chemicals sterilisation, and is still readily available (Holcomb et al., 2022). PETG is slightly more challenging to print with than PLA as it is more prone to oozing, however, these challenges can be eliminated by the fine tuning of 3D printers (Ramírez-Revilla et al., 2022).

ABS was not considered, primarily, due to the difficult printing conditions required, as well as the potential to release toxic fumes during printing (Algarni and Ghazali, 2021).

#### Electronic design

An Arduino nano (ESP32) board (sampling rate 1,000 Hz and ADC resolution 0.805 mV per step) was chosen to acquire the data *via* the MXP5010DP differential pressure sensor. These boards are extremely cost-effective solutions compared with traditional microprocessors, costing just £17.45. Given that they are resilient and have low power consumption, they are ideal for frugal innovation purposes. The circuit for this design would be relatively simple since the sensor only requires three pins to be connected: Vout, which would connect to the analogue input pins in the Arduino; a ground pin; and a 5 V pin. These would be interfaced by a breadboard.

To ensure clear signal processing, signal conditioning circuity was considered and added. This would reduce noise that may interfere with the precise data readings. First, two decoupling capacitors were implemented in the circuit. A 10 µF electrolytic capacitor was placed across the power supply to the sensor to smooth variations in the supply voltage to the sensor, which may cause fluctuations on the sensor pins Vout, and a 0.1 µF ceramic capacitor was placed at the sensor output to suppress high frequency noise entering the analogue pin.

This was necessary as the sensor had a short response time of 1 ms meaning that data was collected every 1 ms, resulting in a sampling frequency of 1,000 Hz. The ATS/ERS spirometry technical standards (Graham et al., 2019) state this should be at least 100 samples per second (100 Hz).

Furthermore, the sensor also had a typical max output voltage of 4.7 V, which is much greater than the max input voltage of the analogue pins of the selected Arduino, rated for only 3.3 V. Therefore, a voltage divider was implemented into the sensor pins output to scale down the voltage by a factor of 1.47, using a 4.7 kΩ and a 10 kΩ resistor. These values were chosen as they reduced the maximum voltage entering the Arduino to 3.2 V avoiding saturation and damage to the board. The resistance was low enough to not affect the ADC stability.

The signal was then scaled up and converted in voltages in MATLAB by taking into account the ADC resolution and the initial scaling down factor. A max voltage of 3.3 V, corresponding to 4,095 levels, would then be scaled back up to 5 V using a scaling factor of 1.47.

Additionally, to increase the usability of the design, three LEDs were added to act as indicators for the user as they perform the spirometry test. A red LED to indicate that there is no serial connection between the device and the software, a yellow LED to indicate that the device has established a serial connection and should press the start button to begin data collection, and a green LED to indicate that the user should start exhalation. An LCD screen was used in conjunction with to indicate more specific commands and return spirometry variables at the end.

### Software

**FIGURE 1:**
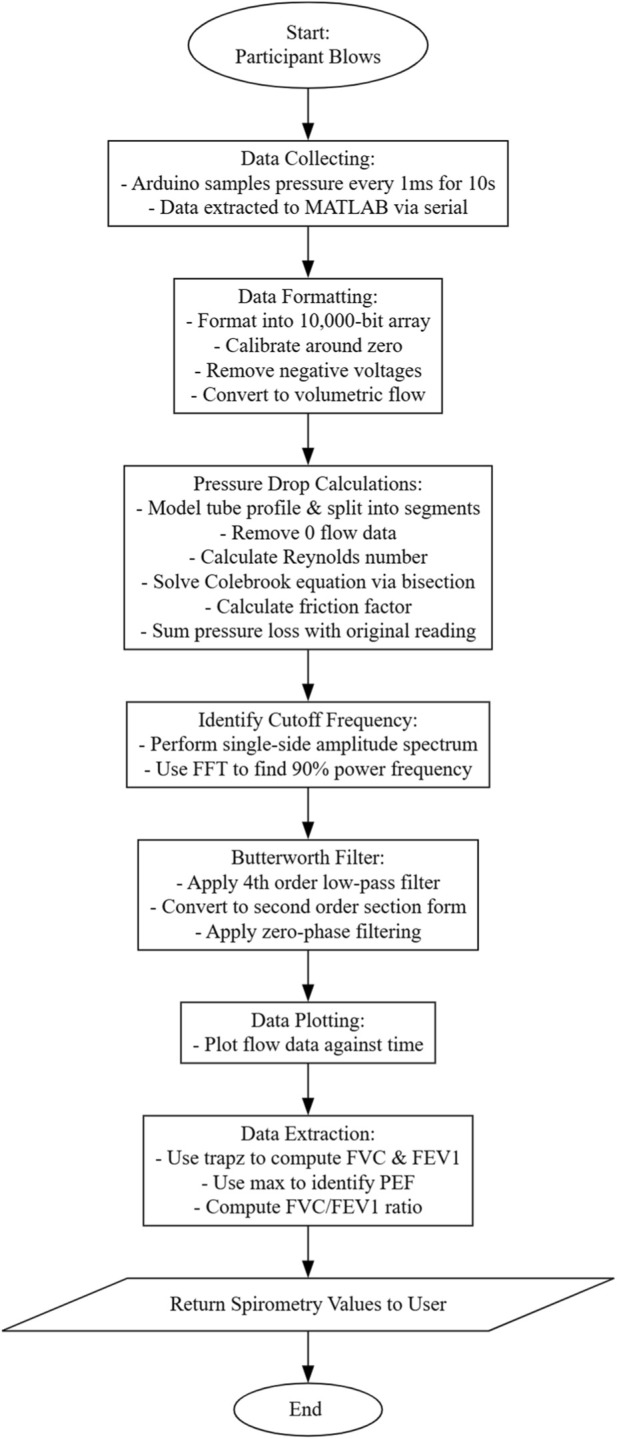
Flowchart of data processing steps within MATLAB software that converts voltage readings to accurate fluid models and therefore spirometry data.

Software was developed to collect and process the data from the hardware and return the spirometry values to the user. To do this, an overall flow diagram was developed to describe the steps from data collection to the return of processed data (Figure 1).

With respect to software, two sets of code were developed, one within the Arduino for reading and transferring data and one within MATLAB for data analysis and display. Using this consistent format, returned spirometry data will always be comparable with those of other tests. Similarly, using the workspace feature associated with MATLAB, trials can be run multiple times, changing input variables within the code to determine the optimum settings for spirometry flow analysis.

### Testing and validation

To test the accuracy of the device and software, two tests were planned, namely, one to measure its accuracy in a standard lab test using a 2-L handpump, and one to perform an initial usability study for the device.

#### Two-litre hand pump test

This test was performed to measure the accuracy of the device at measuring a known volume of air. To do this, the 2-L handpump was connected to the manufactured spirometry tube *via* a 3D-printed friction-fitted attachment that was designed to eliminate air loss between the pump and the tube. The tube was secured firmly to a table so that it lay horizontally. This eliminated possible fictitious readings that could be generated by moving the tube through the air; similarly, objects behind the device were removed to prevent any reflections of air back into the device.

With the experimental setup complete, the handpump was fully extended, and the MATLAB program was run. Moreover, the pump handle was compressed until the air was fully expelled from the handpump through the device. The experiment was run 30 times at varying speeds, and the data were saved and recorded for later analysis after each trial. The varying speed helped us investigate whether the flow had a significant influence on the accuracy. This variable would also verify whether the drag equations had been modelled correctly, since the PEF is correlated with drag. The speed range investigated would be hard to determine since this was calculated after the test. However, the duration over which the full 2 L could be monitored and recorded and would correlate to the flow rate. The range of duration investigated was from 0.3 to 2.5 s. The length of duration was monitored and recorded in MATLAB, by assessing the length on the time axis of the flow rate time graph that was above the threshold value during expiration.

The accuracy was calculated *via* the mean percentage error. Moreover, further comparisons between the signed percentage error (SPE) and the duration of each blow were made to investigate whether the speed of the input flow affected the accuracy. As a first step, the normality of the data was tested with the Shapiro-Wilk test (Shapiro and Wilk, 1965), with a p-value lower than 0.05 indicating a non-normal distribution. The variable distribution proved to be non-normal (W = 0.6758, p-value < 0.001). Subsequently, Spearman’s rho (Schober et al., 2018) and its p-value were calculated. P-values lower than 0.05 were considered significant.

#### Prospective evaluation study

The second test assessed the viability of the device as a spirometer, achieved by comparing the device against a benchmark CE marked spirometer. For this purpose, the test involved recruiting 30 participants and asking them to simulate the use of the spirometer after receiving instructions.

The sample size was determined by an *a priori* power analysis conducted using the G*Power tool to ensure sufficient statistical power for the primary aim of comparing the prototype against the reference device (Kang, 2021). The analysis was based on a bivariate normal model correlation test, with an alpha of 0.05, a desired power of 0.9, and effect size parameters set to a null hypothesis correlation (rho H0) of 0.6 and an alternative hypothesis correlation (rho H1) of 0.85, which indicated a required minimum sample size of 30 participants.

The users, whilst sitting, had to take as deep a breath as possible and exhale this air through the tube as quickly as possible, performing the so-called “full forced expiration.” For hygienic reasons, disposable filtered mouthpieces were used (https://www.numed.co.uk/products/disposable-bacterial-viral-filters-for-spirometry-pack-of-50). The participants were asked to repeat this test six times each (with a minimum of a 30 s rest between each blow), however, the first three trials were used as mock tests, so that the user could become accustomed to the device. For comparison, after collection with the developed device, each participant was also asked to perform six forced exhalations through a CE-marked spirometer (MIR SmartOne Bluetooth-to-Phone Spirometer, which collected participant PEF and FEV1, to add further insights and allow comparisons.

The reference CE marked spirometer is designed for home monitoring and, therefore, does not require calibration out of the box. The device, however, is factory calibrated, conforming to ISO 26782 (Standardization IOF, 2009) and ISO 23747 (Standardization IOF, 2009; VanZeller et al., 2019) standards for peak flow and spirometry devices. No calibration was performed to validate this benchmarking device outside of this.

The data from the developed device was extracted by first establishing the peak flow (the maximum data point), and this became a reference point for the other variables. The duration of each exhalation was calculated by finding the time stamp of the peak and iteratively working back through data points until the flow rate was a 4% of the maximum on either side of the peak. This percentage was determined a comparable power to that of the noise of the sensor and is negligible for the total volume. The FVC was found by measuring the total area within the duration and the FEV1 by measuring the total area under the curve within the first 1 s of the duration.

An initial scatter plot of the data was made to visually assess the agreement between devices with mean values from the developed device from each participant on the y-axis and mean values from the comparator on the y-axis. A Shapiro-Wilk test (Shapiro and Wilk, 1965) was performed to test the normality of the data and subsequently either the Pearson or Spearman correlation (Schober et al., 2018) would be evaluated to assess the strength of the relationship, if p-value was found to be lower than 0.05 the data was deemed to be statistically significant. A Bland-Altman plot analysis was performed to evaluate the limits of agreement between devices; helping to identify systematic bias within the device and the range it fell within (Gerke, 2020). Moreover, the ICC was calculated to determine the reliability of the data (Liljequist et al., 2019). Error metrics such as mean absolute error (MAE), mean absolute percentage error (MAPE), mean signed percentage error (MSPE), root mean square error (RMSE) and bias (mean difference) were all calculated so the accuracy could be assessed (Hodson, 2022).

## Results

### Hardware

#### Tube design

**FIGURE 2:**
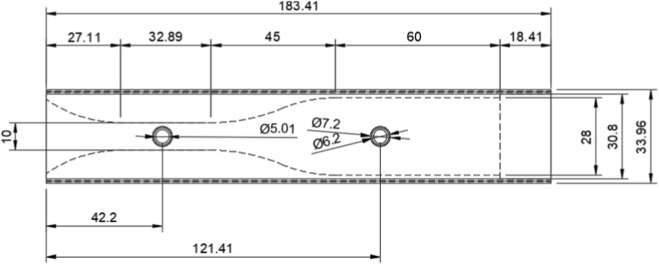
Technical Drawing of the proposed Venturi tube used to create differential pressure units in mm. The Cross sectional analysis of the device is shown, to demonstrate the internal geometry that creates the differential pressure.

**FIGURE 3:**
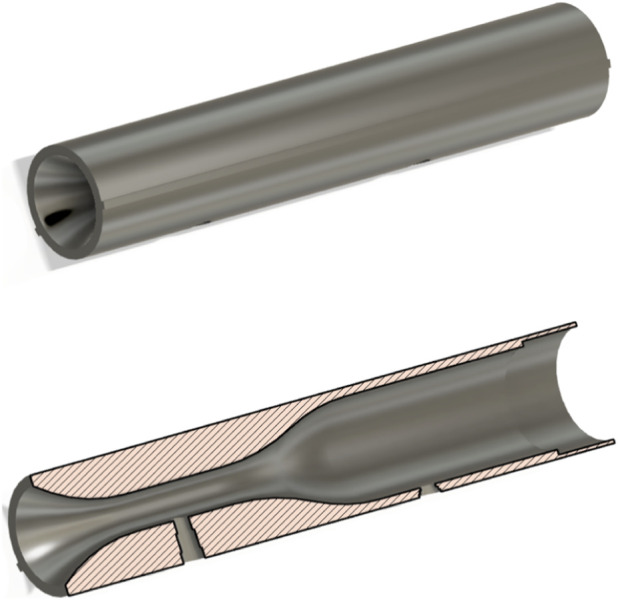
(Top), 3D model of the main spirometry breathing tube developed on fusion 360. (Bottom), shows a spliced cross section of the main tube to demonstrate internal geometry including two ports where differential pressure is measured between.

Based on the boundary condition factors, such as the average PEF of humans (deemed to be 600 L/min (Clinic, 2024)), the inlet diameter of the micro bacterial filter (mouthpiece available at https://www.numed.co.uk/products/disposable-bacterial-viral-filters-for-spirometry-pack-of-50) (28 mm), and the maximum differential pressure range of the sensor (10 kPa), an outlet diameter of 10 mm was deemed suitable for most accurately modelling and collecting data between the two differential pressure points. This diameter would allow for the generation of a maximum of 8 kPa. These diameters were taken in conjunction with Venturi tube specifications from the British Standards Institute, and a model designed on Autodesk Fusion was made (Figures 2, 3). In particular, there is a divergent outlet that smoothens flow dissipation, and a smooth transition region between ports to eliminate unpredictable flow. The two diameters at the inlet allow for the mouthpiece to fit comfortably within the device.

#### Casing

**FIGURE 4:**
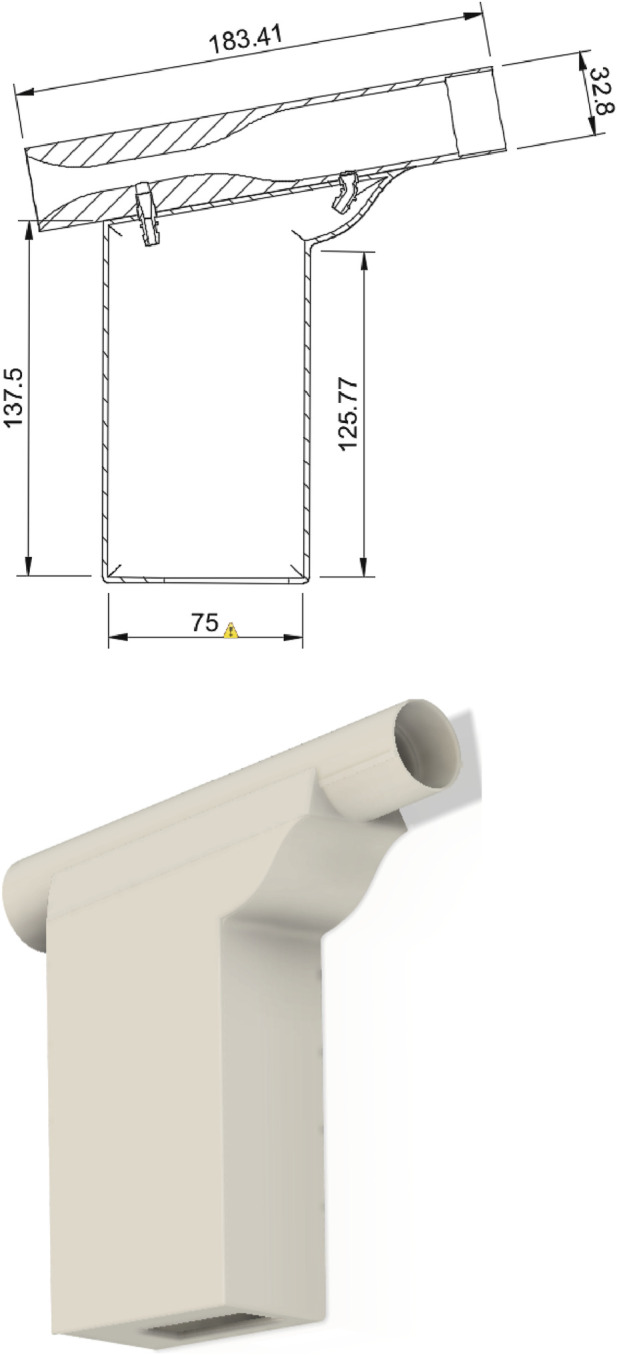
(Top) Technical drawing of the casing including the venturi tube, (bottom) Full assembly of the 3D printed casing. The sizes measures are reported in mm.

The casing was designed to hold electronic components secure relative to the spirometry device so that by moving the tube it would not loosen or weaken electrical component connections. The entire Venturi tube and case were designed to be assembled in five components. The five components include the Venturi tube, the bottom casing, which allows electronics to be fitted and secured; the top casing to enclose the electronics from the elements; and finally, two 3D printed ports which interface between the Venturi tube and the differential pressure sensor. The full assembly is shown in Figure 4.

The device in Figure 4 was designed to reduce the amount of printing material and support material for manufacture. Thus, it was manufactured with five assimilable components rather than one bulk component. This has three benefits. First, within LRS it is important to minimise the precious materials being wasted from support structures, which could be used to make other components. Second, in LRS reliable power supplies are not always guaranteed, therefore by splitting the device into multiple printable pieces it minimises the risk of total print failure in the event of power loss. Finally, orienting the prints individually to reduce material waste ensures a better print quality and reduces rough surfaces left over from the removed support material, which could harbour more bacteria when in use or influence air flow.

The device is assembled by sliding the Venturi tube into the top casing using guide rails to lock the tube orientation in place; then it slides until it reaches inhibitor blocks in the rails. The Venturi ports that interface between the tube and the sensor are then inserted into the device to lock the Venturi tube from sliding. The electronic components can then be placed, and the sensor can be connected to the tube *via* plastic stubbing with an internal diameter of 4 mm. For the device used in the testing and validation shown in Figure 4, the casing exhibits a hole at the bottom of the device to allow a Universal Serial Bus (USB) cable to power and transfer data. In the future, this hole will be removed since a battery and Bluetooth will be used.

This device was 3D printed using an Ultimaker 2+ 3D printer with a 2.85 mm polylactic acid (PLA) filament at a 0.1 mm layer height, a wall thickness of 1.2 mm and an infill density of 40%. The print did not require any support material as it was printed with the truncation of the tube at the bottom of the print. This meant that the layer orientation was aligned perpendicularly with the direction of air flow. This resulted in a wall roughness of approximately 10 μm.

Due to the printer limitations, specifically, with the dimensional tolerancing error generated from the nozzles physical size, the actual internal diameters of the print would be smaller than designed (ideal specifications being 28 and 10 mm). A tolerance of ±0.2 mm was used for the diameters and the resulting true diameters were estimated to be 27.8 and 9.8 mm. This was reflected in the code when declaring variables, with radius’ set to 13.9 and 4.9 mm respectively.

### Software

The Arduino and MATLAB software were designed to interface together so that the MATLAB code could control when the Arduino would collect data from the sensor and when to stop collecting data from the sensor. This was implemented using the serial port so that, when the board detected a new connection, it would start collecting data. Once enough data were collected (10,000 data points lasting over 10 s), this connection was terminated.

As mentioned previously, after setting up initial variables for detection from the sensor, the Arduino enters a wait mode where it waits for a serial connection to be established by the computer. Until then, no data is collected. Once a serial connection is established, the code starts collecting data *via* the analogue read function from the sensor pin every 1 m. Within this loop, these datapoints are sent to MATLAB *via* serial printing. This process is iterated for approximately 10 s, after which Arduino receives a termination command from the serial connection executed in MATLAB. This causes the device to enter a wait mode again until it is required for the next data collection.

The code operated as expected, as described within the methodology section, following the execution path shown in Figure 1. It successfully interfaced with the Arduino by establishing and disestablishing serial connections with the board to create 10,000-bit arrays of data, computing these into accurate volumetric flows, which could be graphically outputted to find key spirometry values.

### Testing and validation

#### Two-litre hand pump test

**FIGURE 5:**
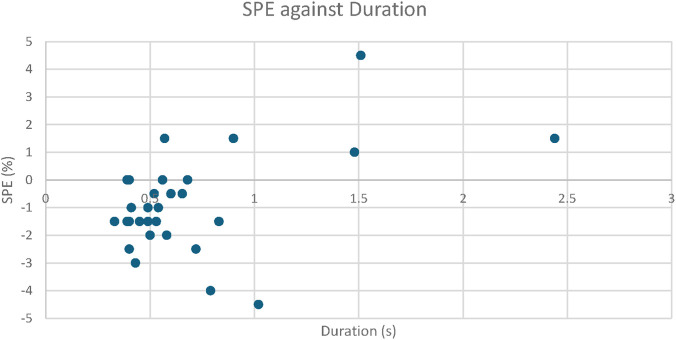
Scatter Plot showing the signed percentage error (SPE) vs. the duration for the 30 trials to measure the 2 L volume.

In the handpump test, the mean value for the measured volume was 1.983 L, whereas the real value was 2 L, and the MAPE was 1.53%, with a mean percentage error of −0.87%. Supplementary Table S1 contains all data related to this. Figure 5 plots the SPE *versus* the duration. The correlation analysis revealed a Spearman’s rho of 0.2717 and a p-value of 0.1464, indicating that there is neither a high nor statistically significant correlation between the input flow and the accuracy of the device.

#### Prospective evaluation study

The results for this evaluation against the benchmark device are reported below. The validation can be split into two categories, comparison between the FEV1 characteristics and the PEF characteristics.

#### FEV1 results

**FIGURE 6:**
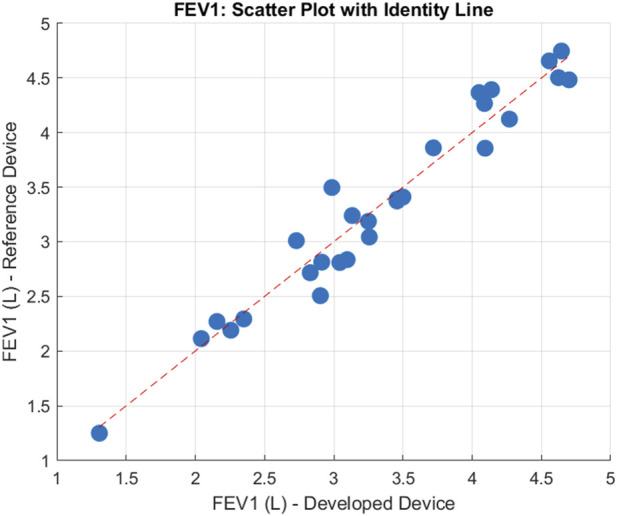
Scatter Plot of the mean FEV1 for 30 participants, compared between both devices. Units are reported in Litres (L).

**FIGURE 7:**
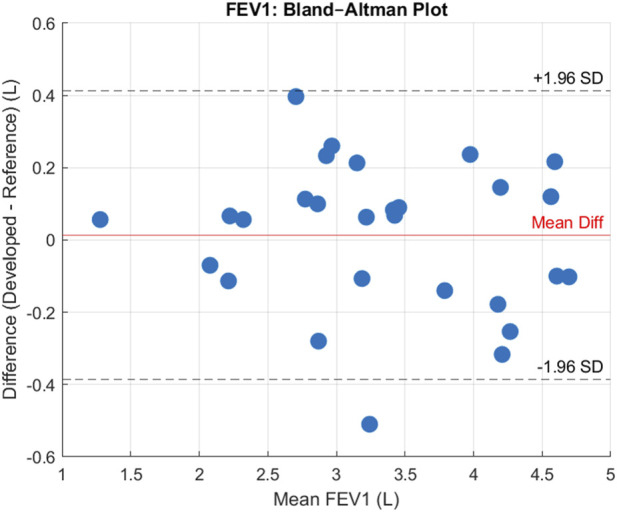
Bland Altman Plot for FEV1 showing level of agreement between devices and bias. Units are reported in Litres (L).

From the 30 participants, comparing measured FEV1 means on each device the following errors were achieved. Our device achieved a MAE = 0.17 L, a MAPE = 5.18%, a MSPE = −0.46% a RMSE = 0.20 L, and a Bias = 0.01 L, suggesting that on average, our device overestimated the volume by 0.01 L. After plotting the data, shown in Figure 6, a Shapiro-Wilks test determined data to be normal and subsequently the data had a Pearson’s correlation *r* value of 0.97, and p-value < 0.001, suggesting there was a statistically significant strong positive correlation for FEV1 between the two devices. The Bland-Altman plot (Figure 7) further supported this showing high agreement, with quite narrow limits of agreement.

The reliability between the two spirometry devices was assessed using the ICC, specifically the ICC(A-1) model. The calculated ICC value was 0.97, indicating very strong agreement. Confidence intervals (CI) were computed at multiple levels: the 99% CI ranged from (0.9327 to 0.9907), the 95% CI ranged from (0.9468 to 0.9881), the 90% CI ranged from (0.9528 to 0.9865), and the 80% CI ranged from (0.9589 to 0.9845). These results demonstrate high consistency between the devices across participants.

#### PEF results

**FIGURE 8:**
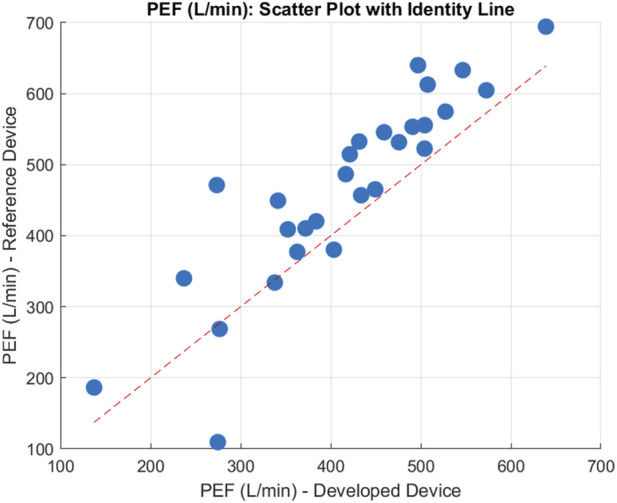
Scatter Plot of the mean PEF for 30 participants, compared between both devices. Units are reported in Litres per min (L/min).

**FIGURE 9:**
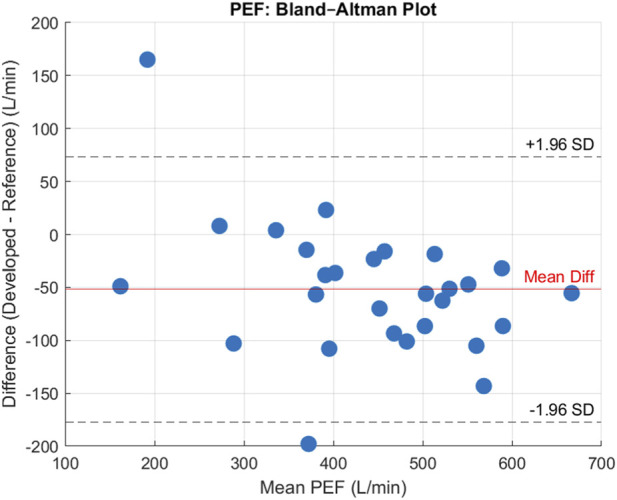
(Top). Bland Altman Plot for PEF showing level of agreement between devices and bias. Units are reported in Litres per min (L/min).

From the 30 participants, comparing measured PEF means on each device the following errors were achieved. The device achieved a MAE = 66.11 L/min, a MAPE = 18.01%, a MSPE = 13.21% a RMSE = 81.34 L/min, and a Bias = −51.80 L/min, suggesting that on average our device underestimated the peak flow rate by 51.80 L/min. After plotting the data, shown in Figure 8 a Shapiro-Wilks test determined data to be normal and subsequently the data had a Pearson’s correlation *r* value = 0.89, and *p* < 0.001, suggesting there was a statistically significant strong positive correlation for PEF between the devices. The Bland-Altman plot (Figure 9) further supported this showing high agreement, with quite narrow limits of agreement.

The reliability between the two spirometry devices was assessed using the ICC, specifically the ICC(A-1) model. The calculated ICC value was 0.80, indicating strong agreement. CI were computed at multiple levels: the 99% CI ranged from (0.5950 to 0.9189), the 95% CI ranged from (0.6538 to 0.8981), the 90% CI ranged from (0.6817 to 0.8860), and the 80% CI ranged from (0.7121 to 0.8706). These results highlight high consistency between the devices across participants.

## Discussion

Based on the results of both the 2-L pump test, which assessed the device’s accuracy, and the evaluation study, which assessed its validity as a lung screening tool, the device’s findings were further compared with standard spirometry testing procedures to determine whether it met the criteria for clinical use.

Diagnostic tools for spirometry are typically calibrated using a 3-L syringe as per the ISO 26782:2009 (Standardization IOF, 2009) guidelines, according to which a mean percentage error difference of 3% is allowed for the measured volume compared with the actual volume. The reason for this is that the threshold set is intended to guarantee reliability when a clinician uses the FEV1/FVC ratio to distinguish between respiratory disease types (obstructive/restrictive). An error less than 3% is considered negligible and does not skew lung parameters enough to affect clinical decision making. In our case, although we used a 2-L pump, we still applied the 3% threshold relative to the measured volume. We assumed the that the percentage error scaled linearly, so that the linear error was proportional to the volume. Based on these assumptions, our measured MAPE of 1.53% remains well within these limits. The main sources of error that could attribute to the percentage error could include factors such as user errors of the pump, filtering errors that lead to over or underfitting of noisy data, limitations of the low-cost sensor (ADC resolution), potential air leakages within the device, tolerance variability within the 3D printed components, environmental factors (temperature, humidity, background airflow) or even specific individuals’ coefficient of variation for each expiration. Whilst this study identifies all these errors listed above, it does not aim to quantify them and should be included in future work.

The performance of this device is comparable to that of other low-cost devices reported in the literature, such as those stated within the introduction. These prior studies that used methods such as differential pressure techniques or other approaches achieved accuracies similar to those of this project. Two Federal Drug Administration (FDA)-approved commercial devices, namely, the AirSmart Spirometer (Ramos Hernandez et al., 2018) and the MIR Spirobank Smart (Ramsey et al., 2021), reported accuracies of ±3% for volume and ±5% for flow, and retail at approximately €69 (Ramos Hernandez et al., 2018) and $190 (Research, 2024), respectively. Additionally, a custom spirometry device developed by Gupta et al. (2011) reported a standard deviation of 8% and a maximum deviation error of 2.5% compared with a standard spirometer. Since our device obtained accuracies similar to those for FEV1 of commercial standard devices, it reinforces its validity as a potential alternative tool in respiratory care. Its production cost is estimated to be approximately £45, covering the Arduino board, differential pressure sensor, 3D printing, and additional electronic components. Large-scale manufacturing could further reduce costs through bulk purchasing.

Compared with the Venturi-based spirometry device by Ferreira Nunes et al. (2024), which was most similar to the device reported in this paper, our device is the result of a frugal, user-driven and contextualised design process, which is paramount for medical devices to be deployed and used effectively in LRSs and LMICs. In terms of performance, their device achieved a MAPE of 1.94% (slightly larger compared to ours of 1.53%) and a SPE of 0.08% (compared to ours which is −0.87%) when a 3 L cylinder was measured. This was achieved using a Venturi-based system (diameter 32 mm constricting to 10 mm), an ESP32 microcontroller and a Honeywell SSCMRRN005PDAA3 differential pressure sensor. Although the full prototype price was not reported, the retail price of this sensor is £74 (Components, 2024), and with this high price, the sensor has a large operating range of −34 to 34 kPa and a %Vfss of 0.25%. Compared with MXP5010DP, which has an operating range of 0–10 kPa and a %Vss of 5%, the Honeywell sensor is technically better. However, within the context of LRS, the NXP sensor is more suitable. Its lower cost of £20 (Components, 2025) makes the device more affordable and accessible, with only a minimal sacrifice in performance.

The MXP5010DP sensor also has several technical advantages in these settings. The NXP sensor is an analogue component that outputs voltage relative to pressure, so it is compatible with any microcontroller, the Honeywell sensor is digital and thus requires a much more precise configuration, requiring a digital communication setup, specialised software and library requirements, and can be damaged easily by supplying the wrong voltage to the sensor. This makes debugging in the LRS more difficult. The Honeywell sensor is factory calibrated in comparison to the NXP device, which requires user calibration to take into account possible voltage offset; however, by allowing user calibration each device can automatically calibrate with the available data making the device more suitable for specific settings, and when the NXP is paired with a suitable low pass filter it can reduce noise to a comparable level to that of the digital sensor. Overall, although the NXP sensor is less technically precise, it still has an error within ±0.5 kPa, which is clinically acceptable for spirometry whilst maintaining several advantages in LRS over the SSCMRRN005PDAA3 (Components, 2025).

Our device differs from the spirometers noted above in its specific design and suitability for LRS. In comparison, it is a fraction of the price at just £45 for base components, and the components used are all affordable and available within LRS. Furthermore, 3D printing can be implemented, and recyclable materials such as PLA can be used. With access to a 3D printer becoming more commonly available, devices can be created locally. This will reduce reliance on imports for components and allow communities to maintain and repair medical devices without external interventions. This was supported by the open-source nature of the project and the design process, which aims to create an easily assemblable device and minimise postproduction processes that lead to increased waste. The device does not have high power requirements and can be connected to either a laptop or smartphone to power the device and interpret data. Future iterations will involve the use rechargeable batteries. Furthermore, the software required does not need continuous internet connectivity to work, supporting use within more rural locations and mobile deployments.

As an alternative frugal spirometry device for implementation in LRS, our device comprises of fewer and more affordable components than its available counterparts do, it incorporates user feedback systems that instruct the user how to use it, and it returns the values of FEV1, FVC and PEF back to the user instantly *via* an LCD screen or *via* the MATLAB script which provides a visual representation of the data. It uses entirely 3D printed components or off-the-shelf bought electronics, so it can be easily replicated in LRS. Since the design is simplistic and primarily focusing on the basic needs of low-income and remote areas, while maintaining clinical accuracy and addressing financial barriers, it can, therefore, be considered a good solution to enhance respiratory care in LRS.

The clinical implications are vast as it brings to light unique features that make it more suitable for LRS. The devices’ ability to measure FEV1 and PEF within clinically acceptable error margins supports its use in diagnosing both obstructive and restrictive lung diseases such as asthma and COPD, but at a fraction of the cost of traditional spirometers. The accuracy lies within gold standards for spirometry devices making it a valid alternative (Graham et al., 2019). It also explores a variety of features that make it more suitable compared with other devices, such as its affordability, portability and support of local manufacturing (readily available components and 3D printing). All these factors would boost its usage in LMICs and promote community level screening. The device aims to support a variety of users from healthcare professionals by returning full spirometry curves, to less confident user in the form of the convenient LCD display, which highlights the key spirometry information required at the most basic clinical level for diagnosis. Having these devices at local levels including in both General Practices (GPs), healthcare centres as well as in patients’ homes could better monitor the progression of respiratory diseases. This would shift heavy workloads off healthcare professionals and local people would feel more empowered to look after their own respiratory health. This could potentially reduce long journeys to see respiratory clinicians and better predict severe respiratory events.

### Limitations

While this work proved to have value as an effective spirometry device, it also has limitations. The device was designed to operate in LRS with minimal resources, and as such, the selected components were chosen to reflect this. Therefore, the individual limits of the components tended to be greater because of constraints such as cost. Most notably, the differential pressure sensor (MXP5010P) chosen had the most limitations, which could be attributes to its error. The sensor could measure pressures in the range of 0–10 kPa (i.e., 75 mmHg). The datasheet of the sensor states that its accuracy is ±5% on the full-scale voltage, i.e., 4.5 V, which means ± 0.225 V. This means that, when small values in the lower portion of the sensor range are read, the measurements will be affected more by inaccuracies. For example, with an input of 1 mmHg (0.1333 kPa), the sensor reading without offset is 0.14 V, which is a comparable number to the stated accuracy of the device. Another possible influencing factor is that at very small and very large readings it is possible that the device has a non-linearity error, such that where the device normally reads linearly, at the extremes, this deviation can lead to different results (Lukat, 2025). Finally, the sensor can experience a factor called offset drift. This is when the nominal offset voltage, which is calibrated at the start of each blow, can change over time, affecting the accuracy. All these factors could be attributed to the observations shown in Figure 5 where it can be seen that at longer durations (lower flow rates) there tends to be a greater percentage error. However, there was non-significant correlation between the two variables. These differences may be due to the limits of the sensor itself and to its accuracy, as mentioned above.

Whilst the sensor was selected primarily for its low costs, it was also considered because of its long-term robustness and environmental resilience to LRS, however, both these factors have not been fully tested. The sensor was rated to only have a 5% max error over a range of −10 °C to +85 °C but could operate from −40 °C to +125 °C, but in general has a temperature coefficient of ±0.02 %FS/°C, which is the drift in the output with temperature changes, so it adjusts its output to account for these temperature variations. Typically, the sensor has a very small linearity error of only ±0.2% of the full-scale span (FSS), meaning that the maximum deviation from the ideal straight line for pressure to voltage is only 0.02 kPa, this makes it ideal for calibration as it is very predictable across the full range of pressures. Its hysteresis error is low at 0.1% of FSS, meaning if the differential pressure was increased to 10 kPa then down to 5 kPa, it would only vary by 10 Pa maximum from the original value, suggesting repeated cycles should not significantly affect this deviation (Components, 2025). The long-term stability of the sensor should be considered further, testing continue use over a period of 30 days to quantify this experimentally.

Factors such as the effect of humidity and dust were not considered on the sensor, and more research should be carried out looking at the long-term effect of these environmental factors on the sensor’s performance. All these factors over time could affect the spirometry variables outputted by the device, resulting in biased readings. The device automatically calibrates itself upon every run of the software accounting for specific changes in the baseline voltage, and resolutions concerned are addressed with suitable filtering. As with all devices, regular inspections and calibration are recommended to assess any large changes over time that could significantly affect output variables. In fact, these sensors limitations are true for any kind of sensor.

With respect to the evaluation study, it was observed that several factors could affect air flow within the device and, therefore, the spirometry results. Each participant used a microbacterial filter to prevent the spread of bacteria and viruses within the device, as well as prevent the inhalation of particles from the 3D printed parts. Each of these factors has an impedance resistance to the flow that may affect the flow speed. However, this effect should be minimal as these mouthpieces are designed on purpose for this kind of use. A more prevalent issue was the fit between the joints. Although the device was designed to have a friction-fitted seal, it may not inherently be airtight, especially if, when the device is used, it moves as the person exhales. This could lead to some air not being directed down the spirometry tube. However, this leak should be minimal if not zero. Further design modifications could be implemented to minimise this further (e.g., combination with other types of seals, use of O-rings, etc.).

Finally, since the design uses 3D printed parts, these parts could influence fluid flow within the device. The feature that was considered was the effect of wall drag due to 3D printed layer structures; this effect was mitigated by calculating this pressure drop within the code estimating wall roughness from printer settings. The wall thickness of the structures was increased to 1.8 mm thick to limit the possibility of air permeating through the structure, at lower thicknesses air may escape from flaws within the structure. The software for this project was developed using MATLAB software which would support the ongoing development of prototype software in a maths-based environment as well as some transition to the future smartphone integration. However, it does have issues for the application of LRS. The licenced nature of the software makes it less accessible to non-expert users such as healthcare facilitators in LMICs (Gentile, 2020). Although this will have to be addressed in future iterations, we already have a working version on Python, which is open access. Future iterations will also integrate the software into a user-friendly app which will provide clear guidance for users as well as feedback. This app should be developed so that it is supported by a wide range of technologies (iPhone Operating Systems (IOS), android, laptops) to increase accessibility of these devices. On board processing for the spirometer was considered to keep the system on one device, however, financial constraints limited us to using a less powerful board. The Arduino board was deemed most suitable especially with respects for prototyping, but it does not have enough computational power necessary to perform the fluid modelling software.

The biggest factors that affect LRS (and commonly LMICS) are often harsh environmental factors such as dust, humidity and high temperatures (Piaggio et al., 2021a). PLA although a suitable material for prototyping, may struggle under these conditions as it could deform under prolonged heat exposure, as well as degrade overtime under high humidities (Ramírez-Revilla et al., 2022). Moreover, common sterilisation techniques such as steam sterilisation could degrade its surface and properties (Ramírez-Revilla et al., 2022). These factors were taken into account but were considered negligible for prototyping development as the misuse of the device was unlikely and spare parts are more accessible. The device would interface with the user *via* a disposable micro bacterial mouthpiece, thus sterilisation concerns were also reduced. Furthermore, PLA can be considered as a relatively brittle material and has low impact resistance. This makes it much more susceptible to breakages from being dropped and may crack if placed under repeated loads.

The casing further incorporates multiple holes for researcher accessibility to electronics; however, these could allow dust to enter affecting sensitive components. These features should be addressed in future iteration, by exploring PETG material and incorporation of batteries and Bluetooth accessibility, reducing port access.

Although this device is in its prototype stage, it has to start conforming to regulatory bodies such as the ISO, FDA or the European medical device regulations, and WHO (World Health Organisation) to enable broader adoption. Specifically, standards such as ISO 26782:2009 (Standardization IOF, 2009) must be followed (maximum error of 3% for measuring a 3 L volume), this governs spirometry calibration and performance to increase trust in these devices. The device currently only uses a 2 L volume to measure accuracy, which is not industry standard. Future testing could be performed with the appropriate 3 L syringe to understand whether performance is similar. Furthermore, since it would be classed as a class II medical device (under FDA) (USFaD Administration, 2025), further rigorous clinical trials and performance validation should be continued to support its clinical accuracy claims. WHO further provides a framework for medical devices specific to LMICs (WHO, 2017), and encourages risk-based classification, which should aim to be followed as this might align better with frugal and context specific innovations (Sustainable development goal 3 (SDG 3)). Finally, since the device uses 3D printed plastics, the ISO 18562 series should be assessed for biocompatibility (Standardization IOf, 2024). All devices intended for respiratory use must comply to this, as it looks at particle emissions, volatile organic compounds (VOCs) and leachables that may cause harm over long term exposure. However, all this goes beyond the scope of this project at this time and entails plenty more resources.

Given the greater implications of these devices, this study shows promise in developing a real-world solution to better manage respiratory disease in LRS. By reducing costs, focusing on more open-source designs and orienting the design around the contextualised needs, a more effective device can be produced. This could increase the rates of early diagnosis as well as better monitor disease progression locally.

Future work is to consider includes investigating the robustness of the casing for electronics, shifting towards the use of rechargeable batteries, and Bluetooth monitoring. Greater testing should be conducted looking specifically at testing to ISO regulations (3 L volume), this will provide greater statistical backing to the clinical results. The sources of errors identified in the discussion above, should be individually analysed to quantify their contribution to the total error. This will identify the key areas research to focus on to improve this accuracy. Finally, more data should be collected from a greater number of participants to validate the device further. Within this data collection, there would be a secondary aim to balance the data collection between healthy participants and people with respiratory diseases. This could then be used in future studies to train a machine learning model to help identify different respiratory diseases. By doing so, the device can shift to becoming more of clinical decision-making tool, which would help less skilled user determine the appropriate course of action when dealing with their lung health.

## Conclusion

This study designed, developed and performed an initial validation of a novel and frugal 3D-printed spirometry device aimed at addressing the contextual needs and challenges of LRS that are often neglected in the design of medical devices. Our study explored unique data processing methods to model fluid flow within a 3D-printed constricted spirometry tube.

The spirometer performed as expected, demonstrating high accuracy and reliability across both tests. In the 2-L volume validation, the device achieved a MAPE of 1.53%, well within the ISO 26782:2009 standard. Furthermore, the lack of significant correlation between airflow duration and SPE suggested that the device was able to maintain accuracy across a variety of flow rates and that fluid modelling equations functioned as expected. When compared with a validated spirometer across 30 participants, the device showed excellent agreement for FEV1. It showed minimal bias (+0.01 L), a high correlation (r = 0.97), and a strong ICC of 0.97. The p-value further supported this suggesting a statistically significant correlation for FEV1 across both devices. This indicates that the device reliably captures clinically relevant lung data. PEF measurements, whilst slightly more variable, still showed strong correlation (r = 0.890) and good reliability (ICC = 0.80), however, found the device had a consistent underestimation, with a bias reading 51.80 L/min under. It is noteworthy that some degree of measurement variability is expected due to the intrinsic nature of the experiment, as human participants cannot reliably perform forced expiratory manoeuvres in an identical manner across repeated trials.

Whilst this study is still in its developmental stage, several factors should be addressed going forward. Such as diversifying the participant involvement from healthy subjects to also include clinical populations (COPD, asthma), which will better inform its clinical effectiveness. We have already partnered with local clinicians and academics in Ghana and Nigeria to understand the feasibility of introducing the product in the healthcare pathways of those locations. Its environmental robustness, durability and regulatory compliance were not fully assessed in this study; despite this should be considered further especially for LRS contexts, in some cases it would require plenty of resources and funding, which go beyond the frugal and open innovation concept.

The results suggest that the device has potential for use in lung function monitoring, particularly in inaccessible spirometry settings and demonstrates the implications that frugal engineering could have, when applied to medical devices. It specifically addresses the challenging environments that often hinder resource constrained settings and enables designs to adapt to a diverse range of contexts so that they are effective and sustainable. This study further underpins that by considering only the required contextual needs, devices can be developed at fraction of the cost without influencing accuracy.

Overall, by developing and adopting more frugal devices, equitable access to respiratory diagnostics can be better achieved in underserved communities. This provides better access to healthcare for LMICs’ and places less reliance upon HICs for donations or aid. Furthermore, the developments for LMICs can also benefit HICs as they address problems uniquely that might not have been identified. This could lead to the adoption of context designed devices into HICs, especially within more impoverished areas within HICs. Whilst further work is needed, this work lays the foundation for local respiratory diagnostics that could benefit both LMICs and underserved populations through reverse innovation.

## Data Availability

The raw data supporting the conclusions of this article will be made available by the authors, without undue reservation.
