# Supplementary material for: A frugal arduino-based spirometer for low-resource settings: design, development and validation of a preliminary prototype
Source: Front Bioeng Biotechnol. 2026 Jan 6;13:1664127. doi: 10.3389/fbioe.2025.1664127 (PMC12816365; doi:10.3389/fbioe.2025.1664127)
Supplement: Supplementary file 1 [file Table1.docx]

Table 1. Results from the two-litre hand pump validation test

| **Trial** | **Total volume (L)** | **PEF (L/min)** | **Duration (S)** | **Signed percentage error (%)** |
| --- | --- | --- | --- | --- |
| 1 | 1.94 | 475.78 | 0.43 | -3.0 |
| 2 | 1.99 | 400.14 | 0.52 | -0.5 |
| 3 | 1.98 | 391.37 | 0.54 | -1.0 |
| 4 | 1.96 | 432.78 | 0.50 | -2.0 |
| 5 | 1.97 | 441.93 | 0.49 | -1.5 |
| 6 | 1.92 | 280.93 | 0.79 | -4.0 |
| 7 | 1.99 | 320.96 | 0.60 | -0.5 |
| 8 | 1.99 | 381.41 | 0.60 | -0.5 |
| 9 | 1.99 | 329.8 | 0.66 | -0.5 |
| 10 | 1.95 | 249.82 | 0.72 | -2.5 |
| 11 | 2.03 | 387.24 | 0.90 | 1.5 |
| 12 | 2.00 | 389.94 | 0.56 | 0.0 |
| 13 | 2.03 | 471.43 | 0.57 | 1.5 |
| 14 | 2.09 | 109.82 | 1.51 | 4.5 |
| 15 | 2.00 | 618.00 | 0.40 | 0.0 |
| 16 | 2.00 | 599.00 | 0.39 | 0.0 |
| 17 | 1.96 | 348.98 | 0.58 | -2.0 |
| 18 | 2.00 | 421.34 | 0.68 | 0.0 |
| 19 | 1.91 | 180.04 | 1.02 | -4.5 |
| 20 | 1.97 | 396.26 | 0.53 | -1.5 |
| 21 | 1.98 | 600.35 | 0.41 | -1.0 |
| 22 | 1.97 | 544.07 | 0.45 | -1.5 |
| 23 | 1.97 | 281.02 | 0.83 | -1.5 |
| 24 | 2.02 | 112.33 | 1.48 | 1.0 |
| 25 | 2.03 | 72.97 | 2.44 | 1.5 |
| 26 | 1.95 | 602.91 | 0.40 | -2.5 |
| 27 | 1.97 | 496.50 | 0.39 | -1.5 |
| 28 | 1.98 | 576.73 | 0.49 | -1.0 |
| 29 | 1.97 | 627.03 | 0.40 | -1.5 |
| 30 | 1.97 | 678.35 | 0.33 | -1.5 |
| *L: Litres*  *L/min: Litres per minute*  *S: Seconds*  *%: percentage* | | | | |

*Table showing the measured forced vital capacity (FVC in L), peak expiratory flow (PEF L/min) and duration of each expiration in (s) after each expiration. The signed percentage error compares the measured volume with the expected volume of 2 litres and is the signed percentage difference between these two values.*
